# Modified unilateral biportal endoscopic transpedicular discectomy for highly migrated upper lumbar disc herniation: a case report

**DOI:** 10.3389/fsurg.2026.1781307

**Published:** 2026-03-25

**Authors:** Yang Wang, Jinming Han, Bingke Zhu, Liujun Zhao, Oujie Lai

**Affiliations:** 1 Department of Spine Surgery, Ningbo No.6 Hospital, Ningbo, China; 2 Ningbo Clinical Research Center for Orthopedics, Sports Medicine & Rehabilitation, Ningbo, China

**Keywords:** migrated disc herniation, minimally invasive spine surgery, transpedicular approach, unilateral biportal endoscopy, upper lumbar disc herniation

## Abstract

Upper Lumbar Disc Herniation (ULDH) is a rare form of lumbar disc herniation, but its symptoms are more severe due to the conus medullaris and the tight spinal canal. Surgical intervention for these fragments is also considered demanding once they demonstrate high-grade downward migration into the “hidden zone” medial to the pedicle. Obstructions due to bone or dangers of damaging nerve tissue by retraction are common limitations of the classical minimally invasive methods. We report on the case of a 69-year-old man with an extreme down-migrated L1–L2 disc herniation (Lee classification Zone 4). The patient had severe left thigh pain and numbness and was unable to walk, with preoperative VAS and ODI scores of 7/10 and 74, respectively. A modified posterior transpedicular approach utilizing Unilateral Biportal Endoscopy (UBE) was successfully conducted. The use of the medial cortical wall of the pedicle as a natural navigational “Cortical Guidance” guides a direct path to the ventral pathology through minimal bone removal and results in true “Zero-Retraction” of the neural structures. Herniated fragments were totally resected postoperatively and the discectomy patient showed significant symptomatic improvement with VAS and ODI scores reduced to 1/10 and 12, respectively. At the 6-month follow-up, the patient had complete neurological recovery. The modified posterior transpedicular UBE technique is a safe, simple, and effective approach for the treatment of highly migrated ULDH and enables extensive decompression with maximal preservation of spinal stability.

## Introduction

Upper Lumbar Disc Herniation (ULDH), characterized as herniations occurring at the L1–L2 and L2–L3 levels, represents a small minority of lumbar disc herniations (1%–5%), but its clinical symptoms tend to be disproportionately more severe and complex. Certain anatomic features in this area, such as the relatively small spinal canal containing the conus medullaris or cauda equina (both are very mechanically compression-sensitive) and a restrained normal range of motion, may contribute to the more profound neurologic deficits patients experience when compared with those presenting with lower lumbar disorders (1–3). Surgical treatment is even more difficult when the herniation develops into a very high-grade downward migration (Lee classification Zone 4) (4), the sequestered nucleus is torn laterally beyond the midline of the caudal pedicle, and it is trapped in the “hidden zone” medial to the pedicle (4, 5).

For such pathologies, traditional open posterior surgeries, such as total laminectomy, afford adequate decompression under direct visualization; however, the extensive disruption of the posterior spinal stabilizing structures carries a high risk of inducing iatrogenic instability and intractable postoperative back pain. This is especially problematic in the upper lumbar area, which has a unique biomechanical environment (6). Percutaneous Endoscopic Lumbar Discectomy (PELD) has become more common in recent years because it is less invasive. However, traditional PELD methods face significant anatomical challenges when dealing with very high-grade, downward-migrated discs in the upper lumbar spine. Specifically, the standard transforaminal approach is often blocked by the bony structure of the caudal pedicle, which makes it difficult to safely and effectively reach lesions located below the pedicle. To circumvent this impediment, surgeons often resort to employing exceedingly steep puncture angles or undertaking extensive foraminoplasty procedures. These actions not only substantially elevate the probability of damage to the exiting nerve root but also frequently culminate in incomplete decompression, a consequence of limited working space and visualization (7–9). While certain researchers have explored the Unilateral Biportal Endoscopy (UBE) technique via a contralateral translaminar approach to circumvent the facet joint, any maneuver necessitating the retraction of the dural sac to reveal ventral pathologies within the constricted spinal canal of the upper lumbar spine presents a considerable risk of catastrophic injury to the delicate conus medullaris or cauda equina (10).

This report presents a case of a patient with a highly migrated ULDH. Following a challenging preoperative diagnostic workup, a definitive diagnosis of a highly migrated L1–L2 disc herniation was established. We successfully treated the patient utilizing a modified posterior UBE transpedicular approach. This technique facilitated complete resection of the extruded nucleus pulposus with minimal bone sacrifice and, crucially, avoided retraction of the neural elements at the corresponding segment. The patient achieved a full recovery with no residual neurological symptoms postoperatively. Written informed consent was obtained from the patient for the publication of their clinical details and accompanying images.

## Case reports

**Figure 1:**
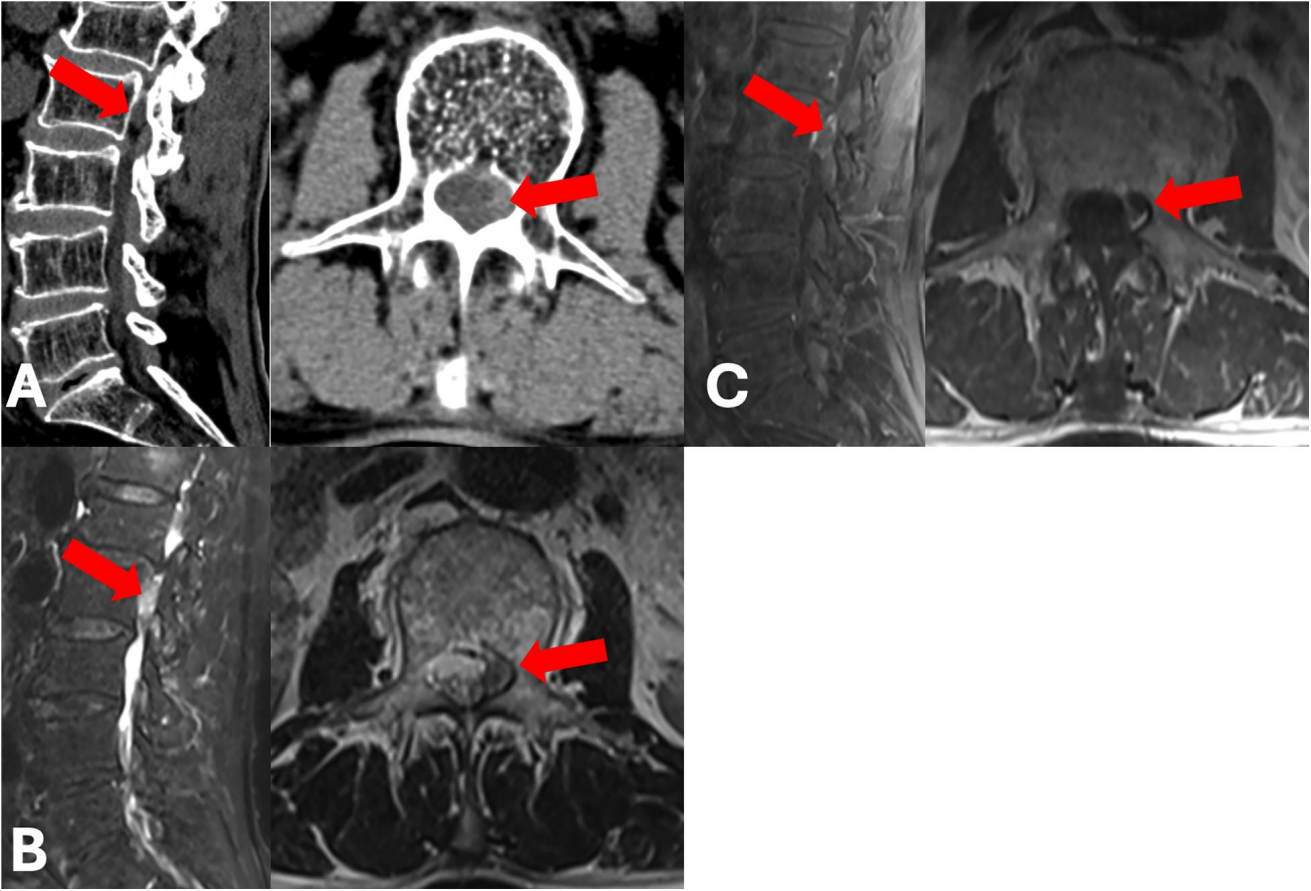
Preoperative imaging studies. The herniated disc is indicated by red arrows. **(A)** Preoperative computed tomography (CT) scan. **(B)** Preoperative magnetic resonance imaging (MRI) scan. **(C)** Preoperative contrast-enhanced MRI scan.

A 69-year-old man, who had smoked for 45 years and had a 10-year history of high blood pressure, came to the hospital with back pain that had lasted for six years. Three months before he was admitted, he suddenly felt pain and numbness in the front of his left thigh after lifting something heavy. Even though he had been treated with non-steroidal anti-inflammatory drugs (NSAIDs) and physical therapy for three months, his symptoms got worse. The patient said that lying down with his hip bent made him feel much better, but the pain and numbness in the front of his left thigh came back as soon as he stood up. Two weeks before he was admitted, the symptoms became so bad that he couldn't walk, which is why he was hospitalized. A physical exam when he was admitted showed tenderness and pain when the thoracolumbar area was tapped. The patient showed reduced feeling in the front of their left thigh. They held their left hip in a flexed position and couldn't take part in a formal muscle strength test for their upper leg. However, the muscle strength in their lower left leg was normal. The right leg showed no neurological problems. There were no herpes skin lesions in the painful area. Other examination results were normal. x-rays of the lumbar spine, taken from the front and side, and during bending and straightening, didn't show any clear spinal instability, but they did show a smaller disc height at the L1-L2 level. Lumbar three-dimensional (3D) Computed Tomography (CT) identified a faint high-density shadow within the spinal canal medial to the left L2 pedicle, though it was inconclusive for a definitive diagnosis (Figure 1A). Lumbar Magnetic Resonance Imaging (MRI) confirmed an abnormal signal adjacent to the inner wall of the L2 pedicle, but as the signal was visible on only a single slice, the diagnosis remained ambiguous (Figure 1B). Given the atypical imaging presentation, the differential diagnosis included intraspinal neoplasms or an epidural hematoma. Since the patient's clinical history and physical signs were insufficient to definitively exclude these etiologies based solely on non-contrast imaging, further investigation was required. To further clarify, a contrast-enhanced lumbar MRI was performed, which ultimately established the diagnosis: a severely down-migrated L1–L2 disc herniation causing compression of the L2 nerve root (Figure 1C). Preoperative VAS and Oswestry Disability Index (ODI) scores were 7/10 and 74, respectively. The patient underwent a modified posterior transpedicular approach using UBE; the detailed surgical procedure is described in the following section. Intraoperatively, fragmented nucleus pulposus was observed distributed along the L2 nerve root, predominantly on its ventral side. The disc fragments were excised incrementally, resulting in the complete decompression of the L2 nerve root. Following the procedure, the VAS and ODI scores diminished to 1/10 and 12, respectively. Muscle strength in both lower extremities was assessed as grade 5, and sensation in the anterior left thigh was restored. The patient indicated persistent pain localized to the surgical incision site. At six-month follow-up appointment, the neurological status of his lower limbs remained exceptional, and the incisional back pain had notably lessened. The overall clinical outcome was classified as “Excellent”.

### Surgical technique

**Figure 2:**
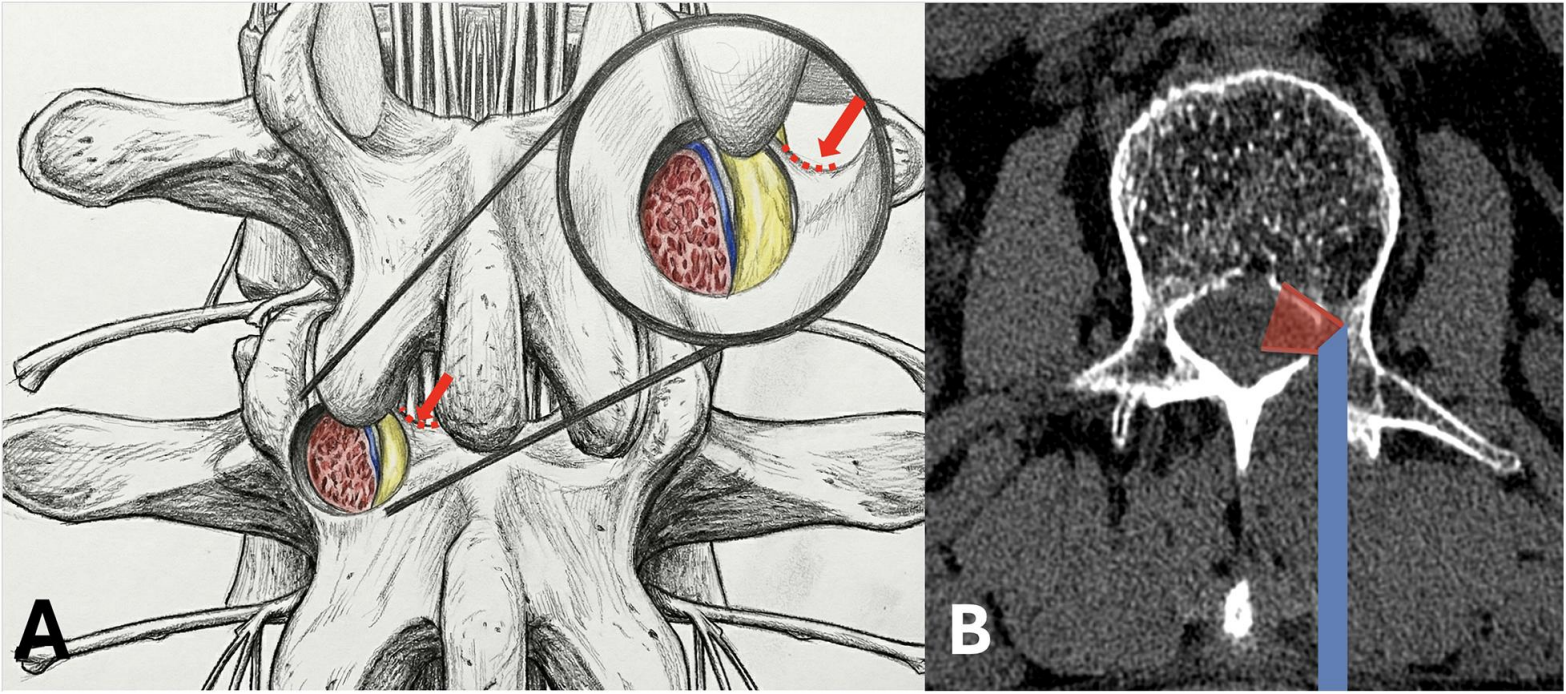
A diagram showing the surgical method. **(A)** A partial bone removal on the lamina exposes the medial cortex (blue) and the cancellous bone (red) of the pedicle, revealing the nucleus pulposus (yellow) located medial to the pedicle. The red arrow indicates the anatomical landmark referred to as the “corner” during the endoscopic procedure. **(B)** The endoscope is then inserted into the medial side of the pedicle to reach the back of the vertebral body and the front of the nerve root. The red area shows the view seen through the endoscope.

**Figure 3:**
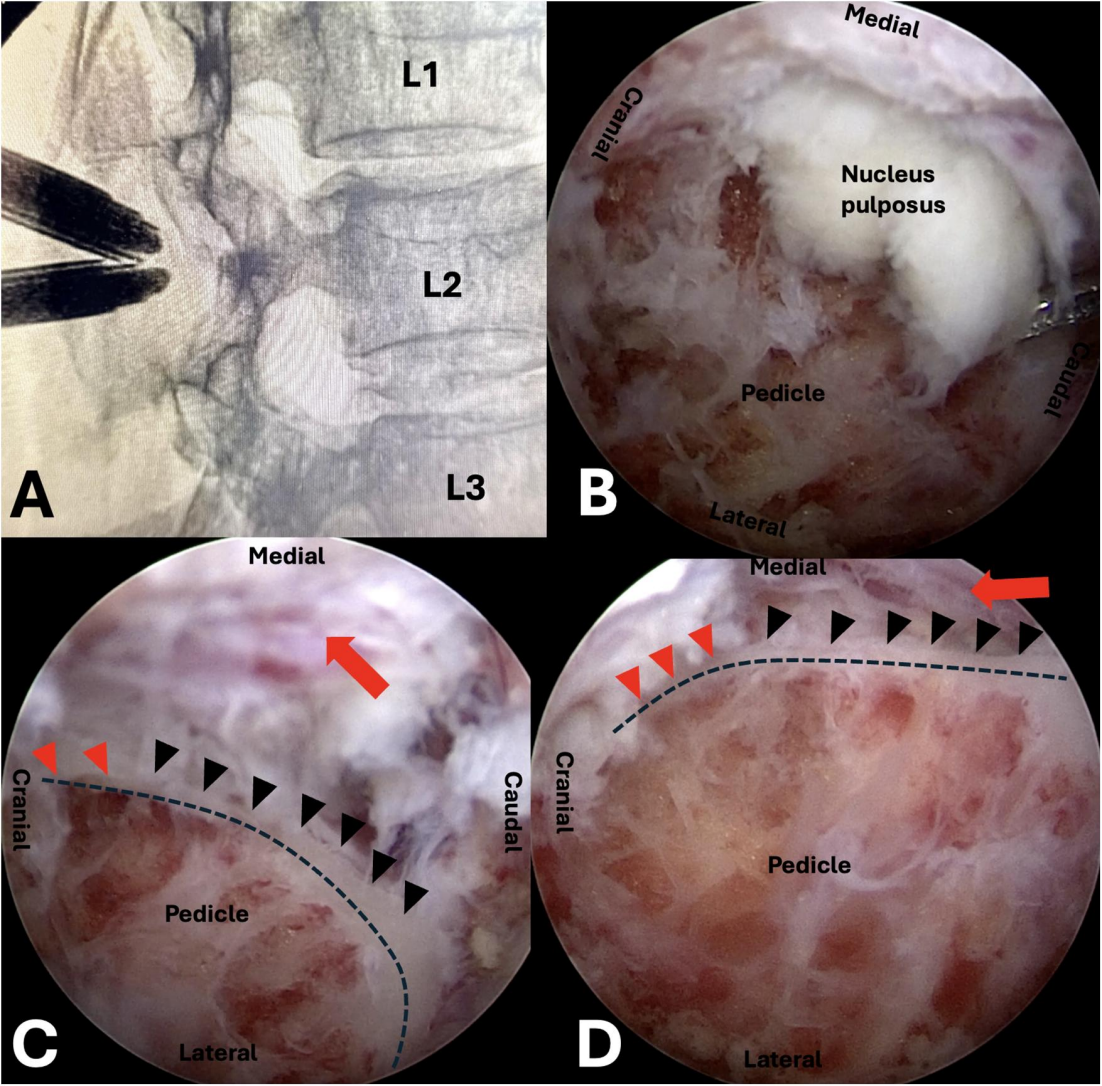
Intraoperative observations. The medial cortex of the L2 pedicle is marked with black triangles, while the cranial cortex of the L2 pedicle is marked with red triangles. **(A)** Intraoperative localization image. **(B)** Intraoperative endoscopic view showing the sequestrated disc fragment (red arrow) located medial to the pedicle. **(C)** Intraoperative endoscopic view showing the decompressed nerve root (red arrow). **(D)** Intraoperative endoscopic view from a different perspective showing the decompressed nerve root (red arrow).

**Figure 4:**
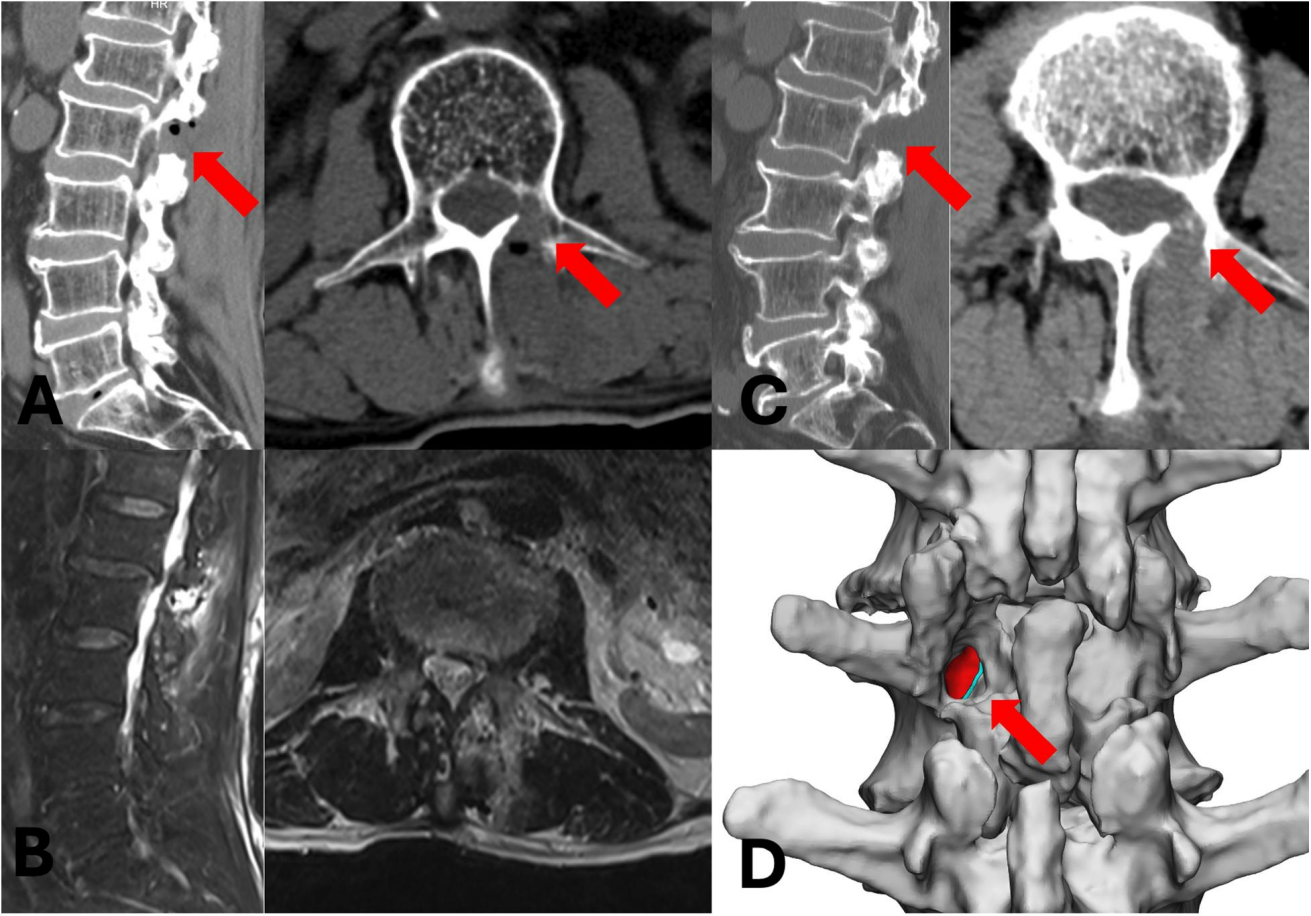
Postoperative radiological imaging. **(A)** Postoperative day 2 CT scan; the red arrow indicates the area of bone resection. **(B)** Postoperative day 3 MRI showing complete removal of the sequestrated disc. **(C)** Follow-up CT scan at 6 months postoperatively; the red arrow indicates the area of bone resection. **(D)** Three-dimensional (3D) CT reconstruction on postoperative day 2. The red area represents the cancellous bone of the pedicle, the blue strip-like area represents the medial cortex of the pedicle, and the red arrow indicates the extent of intraoperative bony decompression.

Following the induction of general anesthesia, the patient was placed in a prone position on a radiolucent spinal operating frame, ensuring the abdomen was suspended. Soft cushions were positioned under the patient's shoulders and bilateral anterior superior iliac spines. As UBE surgery requires continuous saline irrigation, meticulous waterproof draping was essential to prevent fluid soaking and subsequent hypothermia. To obtain clear anteroposterior and lateral fluoroscopic views, the operating table was tilted, rather than the C-arm, to align the L1–L2 disc plane perpendicular to the floor and to ensure symmetrical visualization of the pedicles on the anteroposterior radiograph. Biplanar fluoroscopy and a Kirschner wire were utilized to localize the target surgical landmarks: the left L2 pedicle, the initial target point, and the skin incisions. Diverging from conventional posterior UBE techniques, the initial target point for this case was located medial to the left L2 pedicle on the anteroposterior view and posterior to the L2 pedicle on the lateral view, rather than at the typical base of the spinous process. The incisions were made along the midline between the left L1 and L2 pedicles. Two transverse incisions were created approximately 3 cm apart (depending on the patient's body habitus). The cranial portal, measuring about 5 mm, served as the viewing portal (for the endoscope and water inflow). The caudal portal, about 10 mm, functioned as the working portal (for instruments and water outflow). A blunt dilator was used to penetrate the paraspinal muscles and gently detach them from the lamina. The initial step of the procedure involves triangulating the endoscope (a 30-degree arthroscope) and the surgical instrument at the target point. A radiofrequency wand was employed to clear the soft tissues from the lamina surface and coagulate any bleeders. The exposure was extended laterally to the L2 isthmus and cranially to the transitional “corner” of the L2 superior articular process and lamina. It is worth noting that in the upper lumbar spine, this “corner” may appear less distinct due to a smoother anatomical transition compared to lower levels. However, this surface landmark serves only as an approximate initial reference. The definitive navigational guide is the medial cortical wall of the pedicle located deep to the lamina, which remains distinct and reliable regardless of the surface anatomy's subtlety. Subsequently, a positioning pin was placed approximately 5 mm inferolateral to the critical anatomical “corner,” and its position medial to the L2 pedicle was confirmed with anteroposterior fluoroscopy. Using a 3.5 mm coarse diamond burr, the lamina was gently drilled through until the medial cortical wall of the left L2 pedicle was exposed. This cortical bone serves as a natural guide, with its medial aspect facing the spinal canal and its lateral aspect containing the cancellous bone of the pedicle. The burr was advanced along this medial pedicular cortex cranially to the inferior edge of the L1–L2 disc and caudally to the inferior edge of the left L2 pedicle. This technique, guided by the pedicle's inner cortex, minimizes the need for frequent fluoroscopic checks. Endoscopically, the ruptured nucleus pulposus was visualized ventral to the L2 nerve root, displacing the root dorsally. After removing a small amount of the medial lamina with the burr to better expose the L2 nerve root, a nerve hook was used to release adhesions. Figure 2 illustrates the detailed surgical anatomy. The disc fragments were then hooked and removed piecemeal from along the trajectory of the L2 nerve root. As the fragments were extracted, the L2 nerve root was observed to gradually descend and relax against the posterior wall of the L2 vertebral body. Details of the intraoperative endoscopic images are provided in Figure 3. The dead space was probed to ensure no residual fragments remained. After achieving meticulous hemostasis, a drainage tube was placed to prevent postoperative hematoma. The drainage tube was inserted through the “working portal”. Under endoscopic visualization, it was positioned on the cortical bone surface of the lamina to prevent direct contact with the nerve root, thereby avoiding nerve root irritation or injury. The tube was removed on the second postoperative day, as the drainage volume was approximately 10 mL. Figure 4 demonstrates complete removal of the nucleus pulposus postoperatively.

## Discussion

ULDH, particularly when it exhibits significant caudal migration into the “hidden zone,” presents a considerable surgical obstacle (1, 4, 5). The intricate anatomical features of this condition demand an operative strategy that guarantees thorough decompression while avoiding iatrogenic instability and neural damage. Although conventional open laminectomy affords broad access, its inherent disruption of posterior stabilizing elements frequently results in adverse long-term consequences, including persistent pain and instability (11, 12). Consequently, this has prompted the development of various minimally invasive spine surgery (MISS) techniques, each possessing distinct advantages and disadvantages. Within this framework, our modified posterior transpedicular approach utilizing UBE presents a novel solution, which we will subsequently compare to existing MISS alternatives. PELD, the most frequently employed minimally invasive spine surgery (MISS) technique, offers the benefit of direct decompression of the nerve root from its ventral aspect, thus minimizing manipulation of the susceptible traversing L2 nerve root (13, 14). Nevertheless, as demonstrated in our case, a significant anatomical impediment arises: the superior articular process (SAP) and the pedicle obstruct the pathway to the caudally migrated fragment. To address this challenge, various modifications have been introduced. A common approach involves aggressive foraminoplasty or pediculoplasty, entailing the resection of a substantial portion of the SAP and pedicle to enlarge the foramen (5, 8), which risks damaging the facet joint, possibly causing instability and harming the exiting nerve root. Furthermore, after using the trephine to shape the area, the endoscope's movement is limited. If the removed fragment isn't complete, smaller fragments may remain in the lower part of the spine. This could require foraminoplasty at the nearby level, which would increase surgical trauma and the chance of complications. Other surgeons have also described a full-endoscopic transpedicular approach, similar to the one we used (6, 7). Conversely, the use of a trephine for pedicle shaping in this method presents a significant potential for iatrogenic damage to the upper lumbar spine's intracanal structures. Furthermore, the single-portal system is subject to inherent constraints, including instrument “sword-fighting,” a steep learning curve, and difficulties in achieving hemostasis, which may result in residual disc fragments. An alternative PELD-based approach, the “Vertebral Trench Technique” (VTT), involves creating a channel on the posterior vertebral body to circumvent the pedicle and access the migrated fragment (9). While this design is innovative, it is technically challenging, poses a risk of bleeding from the vertebral body's cancellous bone, and is primarily applicable to upwardly migrated cases, rendering it inappropriate for the pathology observed in our case.

Due to the difficulties in reaching certain highly migrated disc fragments through the lateral corridor, posterior approaches have been explored by surgeons. As a result, translaminar and contralateral approaches have been suggested. The classic translaminar “keyhole” technique is very effective for upwardly migrated discs and could theoretically be used for caudally migrated cases. However, these methods often require frequent fluoroscopy to ensure the laminotomy's position is accurate, which leads to a relatively higher radiation dose. Furthermore, posterior approaches usually require the nerve root to be pulled medially to expose the ventrally located fragment, a maneuver that poses a significant risk in the upper lumbar spine (1, 15, 16). The UBE contralateral sublaminar approach provides good access to the “axillary” or “shoulder” region of the contralateral nerve root, but the maneuver across the dural sac may not be suitable for ULDH cases (10, 17). This is particularly critical in the upper lumbar spine, where neural elements are more vulnerable to fluctuations in hydrostatic pressure and mechanical manipulation. Furthermore, given the relatively small size of the herniation in this case, a contralateral approach would necessitate disproportionate bone resection, thereby increasing the risk of iatrogenic instability. In contrast, the transpedicular technique described herein offers a shorter, more direct trajectory to the lesion, making the procedure simpler and more efficient.

Our refined posterior transpedicular UBE technique aimed to combine the benefits of a direct corridor with the improved ergonomics of a biportal system. Its distinct value, in comparison to the previously mentioned methods, is readily apparent. Specifically, the technique's enhanced safety is achieved through “Cortical Guidance,” a crucial technical element that utilizes the medial cortical wall of the pedicle as a natural guide and protective barrier. By carefully drilling along this cortical surface, we create a safe path. This prevents accidental entry into the spinal canal or excessive removal of the pedicle. Anatomically, as the drilling approaches the medial wall, the cortical bone becomes relatively thick, presenting a distinct demarcation between the cancellous and cortical layers. Visual recognition of this specific endoscopic landmark allows for precise control, ensuring that the burr does not inadvertently breach the canal or injure the neural tissue. This built-in safety feature reduces the need for frequent fluoroscopy and lowers the risk of nerve damage (1, 15, 16).

True “Zero-Retraction” Decompression: The surgical route is created entirely through bone, allowing instruments to reach the front of the nerve root directly, where the fragment is located. This approach completely avoids manipulating or removing the already compressed and inflamed nerve structures, which is a major benefit for encouraging the best possible neurological recovery.

Maximal Preservation of Bony Stability: Using the medial pedicle cortex as a precise reference point, the bone removal is carefully limited to the necessary pathway. This reduces overall bone damage and exposure, significantly lowering the risk of fractures caused by the procedure and preserving the structural integrity of the pedicle and lamina, thus preventing instability after surgery. Crucially, a profound understanding of endoscopic anatomy is a prerequisite for this procedure. It is essential for precisely locating the pedicle and avoiding iatrogenic instability caused by excessive bone resection. The pedicle is generally situated caudal and lateral to the “Corner” —defined as the junction between the superior margin of the lamina and the superior articular process. Importantly, the lamina in this area lacks ligamentum flavum attachment. Consequently, by simply removing the lamina in this region, the medial edge of the pedicle can be directly accessed, thereby facilitating true nerve-retraction-free decompression, as detailed in this study.

However, the limitations of this study must be acknowledged. Because it is a single case report, the successful outcome cannot be generalized to a larger group without further evidence. Furthermore, highly migrated disc herniations are classified into upward and downward migrations. While the case described herein involved significant downward migration, the applicability of this technique to upwardly migrated discs remains unverified. Theoretically, however, the concept of “Cortical Guidance” via the pedicle wall could be adapted for highly up-migrated herniations. This would necessitate utilizing the pedicle cranial to the affected disc as the navigational landmark, with the decompression zone primarily situated medial and caudal to that pedicle. This potential application warrants further investigation and study. In addition, there were specific diagnostic and procedural limitations in this case. Regarding the preoperative workup, given that the patient's primary presentation was severe radicular pain rather than motor weakness, we prioritized contrast-enhanced MRI to define the anatomical compression and did not perform electromyography (EMG) to objectively quantify the functional impairment of the nerve root. Furthermore, while intraoperative neurophysiological monitoring (IONM) is a valuable tool for preventing neural injury, it was not utilized in this procedure. Due to institutional resource allocation, IONM is currently reserved for cervical and thoracic surgeries at our center and was unavailable for this lumbar case.

In summary, the modified posterior transpedicular UBE approach constitutes a rational and efficacious advancement in the management of severely down-migrated ULDH. By incorporating a direct transpedicular corridor with the ergonomic and visual benefits of the UBE system, it offers a secure, reproducible, and reliable technique for achieving comprehensive decompression while maximizing spinal stability.

## Conclusion

The surgical management of severely down-migrated ULDH presents a considerable challenge, given that standard minimally invasive methods frequently prove inadequate for achieving sufficient decompression, potentially leading to iatrogenic instability or neural damage. The modified posterior transpedicular UBE approach, which combines a direct transpedicular corridor with the ergonomic and visual benefits inherent in the UBE system, constitutes a rational and efficacious advancement. Notably, this technique diminishes the reliance on heavy intraoperative fluoroscopy. Once the anatomical “Corner” is identified endoscopically, surgeons can easily locate the medial wall of the pedicle situated laterally and trace this cortical bone to safely expose and decompress the nerve root. Consequently, this modified approach offers a secure, consistent, and reliable means of attaining complete decompression while simultaneously optimizing the preservation of spinal stability.

## Data Availability

The raw data supporting the conclusions of this article will be made available by the authors, without undue reservation.
