# Supplementary material for: Modified unilateral biportal endoscopic transpedicular discectomy for highly migrated upper lumbar disc herniation: a case report
Source: Front Surg. 2026 Mar 25;13:1781307. doi: 10.3389/fsurg.2026.1781307 (PMC13057273; doi:10.3389/fsurg.2026.1781307)
Supplement: Supplementary file 1 [file Table1.docx]

**Note regarding Supplementary Video:** Our submission is accompanied by a high-resolution surgical video (>1GB) that demonstrates the key technical steps described in the manuscript. Due to file size limitations, we were unable to upload it via this portal. **We have the file ready for immediate transfer.** Please kindly instruct us on your preferred method for receiving large media files (e.g., secure cloud link, FTP, or email transfer), and we will provide it immediately.

【Modified Transpedicular UBE: Cortical Guidance and Zero-Retraction Decompression】 https://www.bilibili.com/video/BV1gkqCBPEE5/?share_source=copy_web&vd_source=a3e0f34135e45ef0f4c52b236044fe9c
